# Factors Associated With the Nutritional Status of Children and Their Parents: A Cross‐Sectional Analysis With Families Registered in Family Health Units, ES/2021

**DOI:** 10.1155/jobe/3067261

**Published:** 2026-04-27

**Authors:** Kéllyda Cinnara da Silva Moura, Oscar Geovanny Enriquez-Martinez, Haysla Xavier Martins Mendonça, Leticia Batista de Azevedo, Ana Maria Abreu de Oliveira, Ana Luiza Leite Costa, Pedro Antonio Souza de Almeida, Maria del Carmen Bisi Molina

**Affiliations:** ^1^ Department of Nutrition and Health, Federal University of Espírito Santo, Vitória, Espírito Santo, Brazil, ufes.br; ^2^ Health Sciences Center, Federal University of Espírito Santo, Vitória, Espírito Santo, Brazil, ufes.br; ^3^ Department of Pediatric and Adult Clinics, Federal University of Ouro Preto, Ouro Preto, Minas Gerais, Brazil, ufop.br

**Keywords:** family health, pediatric obesity, primary healthcare

## Abstract

**Background:**

Obesity is a major public health challenge, and its prevalence is increasing in most countries around the world and across all age groups.

**Objective:**

This study aims to identify the correspondence and factors associated with the nutritional status of children aged 7–10 years and their parents/guardians registered in Family Health Units in a metropolitan region in southeastern Brazil.

**Methods:**

Cross‐sectional study with a convenience sample was carried out in 26 Family Health Units in four municipalities in the Metropolitan Region of Vitória/ES, between July and December/2021. An anthropometric examination was performed, and sociodemographic and lifestyle data were collected. Nutritional status was classified using body mass index (BMI). Multinominal logistic regression models were tested, adopting *p* < 0.05.

**Results:**

Around 56.5% of children classified as obese had parents/guardians with obesity. A greater chance of being obese was observed in adults aged > 30 years (OR: 2.34; 95% CI: 1.35; 4.06), Black race/color (OR: 2.82; 95% CI: 1.25; 6.33), and self‐perceived health as regular/poor/very poor (OR: 4.12; 95% CI: 2.28; 7.42).

**Conclusion:**

Children’s nutritional status is only associated with that of their parents/guardians, especially when they have obesity. Higher age range, Black race/color, and worse self‐perceived health were associated with obesity in adults. Public policies should be implemented to tackle obesity at all levels, including the promotion of adequate nutrition, self‐care regarding health, and the encouragement of physical activity within the context of primary healthcare.

## 1. Introduction

Obesity is a major challenge for public health, as it is a multifactorial problem that is difficult to resolve [1] and must be tackled in all countries, especially in middle‐income countries, such as Brazil [2, 3]. Between 1975 and 2016, there was a significant increase in the prevalence of obesity in most countries worldwide and across all age groups. Among adult males, this number rose from 31 million to 281 million; among adult females, from 69 million to 390 million; and among children, from 11 million to 124 million [1].

The increasing trend in the prevalence of overweight and obesity in Brazil follows the global scenario. In the National Health Survey conducted in 2019, it was observed that 60.3% of the adult population were overweight, with 62.6% among women and 57.5% among men [2]. Data from the Food and Nutrition Surveillance System (SISVAN), from 2021, showed that approximately 18% of Brazilian children under 10 years of age were overweight, while 19% were classified as obese [3].

The etiology of obesity is complex, influenced by several factors, including genetic ones, as well as the quantity and quality of food and lifestyle, which directly impact nutritional status [1]. One of the main aspects related to lifestyle concerns the environment, especially the family environment. There is evidence that parents can have an important influence on their children’s health behaviors and weight [4].

In a systematic review with meta‐analysis, [5], based on the analysis of 32 studies conducted in 21 countries classified according to national income level between 2002 and 2015, demonstrated that there is concordance between parent and child with obesity, although the strength of this association varied according to the child’s age and the country’s level of development. Furthermore, the review showed stronger associations when children were older, when both parents to present were obese, and in high‐income countries compared to middle‐income countries.

The importance of the family environment in determining nutritional status is evident, as the correlation between parent and child with overweight seems to be present in dysfunctional family relationships, influenced by unfavorable demographic, economic, and social factors [4]. Parents may inadvertently promote behaviors that contribute to overweight and childhood obesity, such as early interruption of breastfeeding, inadequate eating habits, and excessive screen time (ST) [6]. Parents with obesity are more likely to have children with overweight or obesity compared to parents with adequate nutritional status [7].

Considering the importance of the topic and the scarcity of research conducted in the Brazilian population [8, 9], this study can be very useful in supporting the planning and direction of actions for the prevention of obesity in families, especially in primary healthcare [10]. Thus, this study aims to identify the correspondence and factors associated with the nutritional status of children aged 7–10 years and their parents/guardians registered in Family Health Units (FHUs) in a metropolitan region in southeastern Brazil.

## 2. Methods

### 2.1. Study Design and Population

This cross‐sectional analysis used baseline data from the “Prevention of Childhood Obesity in Primary Healthcare (PrevOI),” an intervention study, conducted in the Metropolitan Region of Vitória/ES, Brazil. The study assessed the impact of an obesity prevention program in children aged 7–10 years registered in FHUs across four municipalities (Cariacica, Serra, Vila Velha, and Vitória), where Family Health Strategy (FHS) coverage ranged from 25% to 78%.

An estimated 43,953 children aged 7–10 years were registered in 82 FHU. To calculate the sample for the main study, we considered a significant difference of 10% between the intervention and control groups, with a Type I error of 5% and Type II error of 20%. The minimum sample size was calculated at 286, with 20% added for potential losses. The sample consisted of 469 participants distributed across 26 FHU.

Recruitment was carried out by community health agents and posters in the FHUs. Eligible children (aged 7–10 years) without cognitive, clinical, or physical impairments were included. After excluding children without complete anthropometric assessments or missing signed consent forms, the final sample provided 95% power to detect associations with nutritional status. Although a convenience sample, it was proportional to FHS coverage in the municipalities.

### 2.2. Data Collection

The data collected were between July and December 2021. Anthropometric examinations and questionnaire applications were performed by a trained team of evaluators/researchers, certified by experienced investigators, within the FHUs’ facilities through prior scheduling with participants. Previously validated questionnaires applied in prior studies were used. Interviews with parents or guardians were audio‐recorded after prior authorization, and the information was recorded on the participants’ forms in their native language (Portuguese). All interviews were conducted in Brazilian Portuguese, the participants’ primary language, without the need for translation.

For the anthropometric examination, participants were instructed to have an empty bladder, be barefoot, wear light clothing, and not wear accessories. Height was measured using a portable stadiometer with 0.1 cm precision from the brand AVANUTRI [11]. Body weight was measured with a digital, Bluetooth‐enabled electronic scale with bioimpedance, with a maximum capacity of 150 kg, calibrated by the brand OMRON HBF222 T [11].

Children’s nutritional status was classified based on the 2007 World Health Organization (WHO) growth curves, using z‐score values [12]. Z‐scores were calculated using the WHO AnthroPlus software. Body mass index (BMI)‐for‐age results were categorized into “thinness” (z‐score between −3 and −2), “normal weight” (z‐score between ≥ −2 and +1), “overweight” (z‐score > +1 and + 2), and “obesity” (z‐score > +2 to +3, including severe obesity).

For adults, nutritional status classification followed the 2000 WHO reference [13], using four categories: “underweight” (BMI < 18.5 kg/m^2^), “normal weight” (BMI ≥ 18.5 to ≤ 24.9 kg/m^2^), “overweight” (BMI ≥ 25 to ≤ 29.9 kg/m^2^), and “obesity” (BMI ≥ 30 kg/m^2^). For older adults (age ≥ 60 years), the cutoff points from the Nutrition Screening Initiative were used [14]: “underweight” BMI ≤ 22 kg/m^2^, “adequate weight” BMI > 22 to < 27 kg/m^2^, and “overweight” BMI ≥ 27 kg/m^2^.

Socioeconomic, lifestyle, and health information about the children and guardians were obtained through questionnaires answered in person by the responsible adult (mother, father, or other family member). Among the variables collected about the children were exclusive breastfeeding time, screen use during meals, breakfast habit, school snack practice, school food index, participation in physical/sports activities, means of transportation to school, and daily ST. The following questions were asked:

1. Breastfeeding: “Do you remember how long your child breastfed?” The duration reported was categorized into “< 6 months” and “≥ 6 months.”
2. Screen use during meals: “Does your child usually have lunch and/or dinner while watching television or using a cellphone?” Answer: “yes” or “no.”
3. Breakfast consumption: “Does your child usually eat breakfast?” Answer: “yes,” “no,” or “sometimes.”
4. School snack: “Does your child usually bring a snack to school?” Answer: “yes,” “no,” or “sometimes.”
 A qualitative Food Frequency Questionnaire (FFQ) was used, with seven frequency options for responses, and the School Food Index [15] was applied to determine food quality. Scores were assigned based on consumption frequencies and then categorized into tertiles. A score ≤ 5 indicated low quality, > 5 to 10 intermediate quality, and > 10 high food quality.
5. Physical/sports activity: “Does your child participate in any sports school, team, or sports training (with a coach/teacher)?” [16].
6. Transportation to school: “Does your child use any means of transport to commute from home to school?” Transport types were categorized as “does not need transport,” “on foot,” “by bicycle,” or “motorized vehicle” (including motorcycle, bus, and car).

ST was assessed according to the recommendation from the Brazilian Society of Pediatrics [17], considering adequate ST < 2 h/day and higher than recommended ST ≥ 2 h/day.

Regarding the guardians, information collected included degree of kinship with the child, age, self‐reported race/color, education level, marital status, number of household members, smoking habits, self‐perceived health, and physical activity (PA).

The socioeconomic classification and family income estimation were based on the criteria from the Brazilian Association of Research Companies [18].

PA was assessed using the long version of the International PA Questionnaire (IPAQ) [19] and classified according to the recommendations [20].

### 2.3. Data Analysis

Appropriate statistical analyses were conducted using IBM SPSS Statistics software, Version 21, with a significance level of *p* < 0.05. The chi‐square test was used to identify differences in the proportions of sociodemographic, lifestyle, and health variables, as well as nutritional status (of children and guardians). To assess the concordance between the nutritional status of children and that of their guardians, Cohen’s kappa test was applied, adopting the reference values proposed by the authors in [21].

Multinomial logistic regression models were applied following the methodology proposed by the authors in [22], stratified by the nutritional status of parents/guardians, to identify associated factors. Variables with a *p* value < 0.20 in the bivariate analyses were included in the regression (sex, age group, education level, self‐perceived health, socioeconomic class, and number of household members).

Model 1 was adjusted for socioeconomic variables, and Model 2 was adjusted for all variables included in Model 1 plus a health variable (self‐perceived health). Crude and adjusted odds ratios (ORs) were presented with their respective 95% confidence intervals (95% CIs), adopting a significance level of *p* < 0.05.

### 2.4. Ethical Considerations

This study was approved by the Research Ethics Committee of the Health Sciences Center of the Federal University of Espírito Santo (protocol no 39633320.0.0000.5060/2020). Children signed the Free and Informed Assent Form, and the adults signed the Free and Informed Consent Form.

## 3. Results

The sample consisted of 455 children and their respective guardians. The mean age of the children was 8.55 ± 1.13 years, and the parents/guardians were 39.8 ± 10.3 years. Among the guardians, the majority self‐identified as mothers (78%). Regarding the children’s nutritional status, 41.3% were classified as having normal weight, 18.3% as overweight, and 40.4% as obese; for adults, 20.9% had adequate weight, 35.4% were overweight, and 40.4% had obesity.

The sociodemographic, lifestyle, and health variables were stratified according to the children’s nutritional status. Most children with adequate nutritional status were 10 years old, female, walked from home to school, did not engage in sports training, had been exclusively breastfed for ≥ 6 months, regularly ate breakfast, had an intermediate quality diet, did not bring snacks to school, used screens during meals, and had ST exceeding recommended limits. The majority of their parents/guardians had obesity. A statistically significant association was observed between the nutritional status of the parents/guardians and children (Table 1).

**TABLE 1:** Sociodemographic, lifestyle, and health characteristics according to the nutritional status of children from the Metropolitan Region of Vitória/ES.

Variables	Total *n* (%) (*n* = 455)	Nutritional status of children			*p* value^a^
		Eutrophy *n* (%) (*n* = 188)	Overweight *n* (%) (*n* = 83)	Obesity *n* (%) (*n* = 184)	
Age (in years)					0.586
07	110 (24.2)	48 (25.5)	22 (26.5)	40 (21.7)	
08	111 (24.4)	44 (23.4)	22 (26.5)	45 (24.5)	
09	106 (23.3)	41 (21.8)	14 (16.9)	51 (27.7)	
10	128 (28.1)	55 (29.3)	25 (30.1)	48 (26.1)	
Sex					0.253
Female	238 (52.3)	106 (56.4)	44 (53.0)	88 (47.8)	
Male	217 (47.7)	82 (43.6)	39 (47.0)	96 (52.2)	
Means of travel (home–school)^b^					0.198
No need	24 (5.3)	8 (4.3)	3 (3.6)	13 (7.1)	
On foot	252 (55.6)	116 (62.0)	41 (49.4)	95 (51.9)	
Bicycle	39 (8.6)	12 (6.4)	11 (13.3)	16 (8.7)	
Motor vehicle	138 (30.5)	51 (27.3)	28 (33.7)	59 (32.3)	
Sports training^b^					0.968
Yes	90 (19.9)	38 (20.4)	16 (19.3)	36 (19.6)	
No	363 (80.1)	148 (79.6)	67 (80.7)	148 (80.4)	
Exclusive breastfeeding^b^					0.235
< 6 months	147 (32.3)	66 (35.1)	21 (25.3)	60 (32.6)	
≥ 6 months	300 (65.9)	121 (64.4)	59 (71.1)	120 (65.2)	
No information	8 (1.8)	1 (0.5)	3 (3.6)	4 (2.2)	
Holds breakfast^b^					0.745
Yes	308 (68.4)	134 (71.7)	56 (68.2)	118 (65.2)	
No	68 (15.1)	26 (13.9)	13 (15.9)	29 (16.0)	
Sometimes	74 (16.5)	27 (14.4)	13 (15.9)	34 (18.8)	
Diet quality					0.548
Loud	122 (26.8)	55 (29.3)	25 (30.1)	42 (22.8)	
Intermediate	181 (39.8)	74 (39.3)	33 (39.8)	74 (40.2)	
Low	152 (33.4)	59 (31.4)	25 (30.1)	68 (37.0)	
Take lunch (school)^b^					0.317
Yes	153 (34.0)	72 (38.5)	22 (26.8)	59 (32.6)	
No	226 (50.2)	90 (48.1)	43 (52.5)	93 (51.4)	
Sometimes	71 (15.8)	25 (13.4)	17 (20.7)	29 (16.0)	
Screen use during meal^b^					0.448
Yes	343 (76.2)	137 (73.3)	65 (79.3)	141 (77.9)	
No	107 (23.8)	50 (26.7)	17 (20.7)	40 (22.1)	
Screen time^b^					0.735
Adequate	37 (8.2)	16 (8.6)	5 (6.1)	16 (8.8)	
Higher than recommended	412 (91.8)	170 (91.4)	77 (93.9)	165 (91.2)	
Nutritional status (guardians)					< 0.001
Eutrophy	95 (20.9)	48 (25.5)	16 (19.3)	31 (16.8)	
Overweight	161 (35.4)	77 (41.0)	35 (42.2)	49 (26.7)	
Obesity	199 (43.7)	63 (33.5)	32 (38.5)	104 (56.5)	

*Note:* PrevOI Study (2020/2021).

^a^Chi‐square test.

^b^N different due to lack of data.

The concordance between the nutritional status of the children and their guardians was assessed. The highest concordance was observed in obesity conditions (22.9%; *p* < 0.001) (Table 2).

**TABLE 2:** Concordance between the nutritional status of children aged 7–10 years and their parents/guardians from the Metropolitan Region of Vitória/ES.

Nutritional status of parents/guardians (*n* = 455)	Nutritional status of children (*n* = 455)		
	Eutrophy	Overweight	Obesity
	*n* (%)	*n* (%)	*n* (%)
Eutrophy	48 (10.5)	16 (3.5)	31 (6.8)
Overweight	77 (16.9)	35 (7.7)	49 (10.8)
Obesity	63 (13.8)	32 (7.0)	104 (22.9)

*Note:* PrevOI Study (2020/2021). Cohen’s kappa coefficient = 0.124, *p* < 0.001.

Sociodemographic, lifestyle, and health variables were also analyzed according to the nutritional status of the parents/guardians. The highest proportions were found among individuals aged < 30 years, female, mothers or fathers, self‐identified as Brown or Yellow race/color skin, with a high school education, married, belonging to Socioeconomic classes *D* and *E*, living in households with ≥ 4 people, nonsmokers, with very good or good self‐perceived health, and physically active (Table 3).

**TABLE 3:** Sociodemographic, lifestyle, and health characteristics according to the nutritional status of parents/guardians of children aged 7–10 years from the Metropolitan Region of Vitória/ES.

Parent/guardian variables	Total *n* (%) 455	Nutritional status of parents/guardians			*p* value^a^
		Eutrophy *n* (%) 95 (20.9)	Overweight *n* (%) 161 (35.4)	Obesity *n* (%) 199 (43.7)	
Age					**0.036**
< 30 years	210 (46.2)	33 (34.7)	82 (50.9)	95 (47.7)	
≥ 30 years	245 (53.8)	62 (65.3)	79 (49.1)	104 (52.3)	
Sex					**0.004**
Female	426 (93.6)	89 (93.7)	143 (88.8)	194 (97.5)	
Male	29 (6.4)	6 (6.3)	18 (11.2)	5 (2.5)	
Degree of kinship					0.990
Mother or father	383 (84.2)	80 (84.2)	136 (84.5)	167 (83.9)	
Other	72 (15.8)	15 (15.8)	25 (15.5)	32 (16.1)	
Race/color^b^					**0.004**
White	83 (18.4)	29 (30.5)	22 (13.8)	32 (16.1)	
Black	103 (22.7)	15 (15.8)	34 (21.4)	54 (27.1)	
Brown and Yellow	267 (58.9)	51 (53.7)	103 (64.8)	113 (56.8)	
Education^b^					
Elementary school	163 (36.0)	27 (28.4)	53 (33.3)	83 (41.7)	**0.030**
Middle school	238 (52.5)	50 (52.7)	88 (55.3)	100 (50.3)	
Higher education	52 (11.5)	18 (18.9)	18 (11.4)	16 (8.0)	
Marital status^b^					0.914
Married	317 (70.0)	65 (68.4)	110 (69.2)	142 (71.4)	
Single	85 (18.7)	17 (17.9)	32 (20.1)	36 (18.1)	
Separated/widowed	51 (11.3)	13 (13.7)	17 (10.7)	21 (10.5)	
Socioeconomic class^b^					0.125
A and B	37 (8.2)	6 (6.3)	17 (10.7)	14 (7.0)	
C1 and C2	174 (38.4)	28 (29.5)	64 (40.3)	82 (41.2)	
D and E	242 (53.4)	61 (64.2)	78 (49.1)	103 (51.8)	
Number of people in residence^b^					0.147
< 4 people	131 (28.9)	31 (32.6)	37 (23.3)	63 (31.7)	
≥ 4 people	322 (71.1)	64 (67.4)	122 (76.7)	136 (68.3)	
Smoking*^b^*					0.946
Smoker	91 (20.1)	18 (18.9)	32 (20.1)	41 (20.6)	
Nonsmoker	362 (79.9)	77 (81.1)	127 (79.9)	158 (79.4)	
Self‐perception/health^b^					< 0.001**^a^**
Fair/poor/very poor	185 (40.9)	20 (21.1)	60 (37.7)	105 (530.0)	
Very good/good	267 (59.1)	75 (78.9)	99 (62.3)	93 (47.0)	
Physical activity^b^					0.214
Active	254 (56.4)	50 (53.2)	98 (62.0)	106 (53.5)	
Insufficiently active	196 (43.6)	44 (46.8)	60 (38.0)	92 (46.5)	

*Note:* PrevOI Study (2020/2021). The bold values indicate significance level of *p* < 0.05.

^a^Chi‐square test.

^b^N different due to lack of data.

Factors associated with the nutritional status of parents/guardians are presented in Table 4. In the crude model, the following factors were associated with overweight: age ≥ 30 years, Black, Brown or Yellow race/color skin, and regular/poor/very poor self‐perceived health. After adjustments, these variables remained statistically significant: age ≥ 30 years, Black, Brown or Yellow race/color skin, and regular/poor/very poor self‐perceived health (Table 4).

**TABLE 4:** Factors associated with the nutritional status of parents/guardians of children aged 7–10 years in the metropolitan region of Vitória/ES.

Variable	Nutritional status of parents/guardians							
	Overweight				Obesity			
	Model 1^a^		Model 2^a^		Model 1^a^		Model 2^a^	
	OR	(IC 95%^b^)	OR	(IC 95%^b^)	OR	(IC 95%^b^)	OR	(IC 95%^b^)
Sexo^c^								
Female	1.00	1	1.00	1	1.00	1	1.00	1
Male	1.98	0.74; 5.31	2.66	0.96; 7.35	0.42	0.12; 1.44	0.68	0.19; 2.45
Age group^c^								
< 30 years	1.00	1	1.00	1	1.00	1	1.00	1
≥ 30 years	2.14	1.25; 3.66	2.44	1.40; 4.25	1.86	1.10; 3.13	**2.34**	**1.35; 4.06**
Race/color^c^								
White	1.00	1	1.00	1	1.00	1	1.00	1
Black	3.21	1.38; 7.44	2.86	1.22; 6.70	3.27	1.50; 7.14	**2.82**	**1.25; 6.33**
Brown and Yellow	2.66	1.37; 5.17	2.24	1.13; 4.46	1.86	1.00; 3.46	1.40	0.72; 2.72
Education								
Higher education	1.00	1	1.00	1	1.00	1	1.00	1
Middle school	2.04	0.94; 4.43	1.64	0.72; 3.64	2.47	1.12; 5.43	1.75	0.76; 3.99
Elementary school	2.13	0.93; 4.86	1.70	0.72; 3.98	3.46	1.51; 7.89	2.45	1.03; 5.83
Family socioeconomic class								
A and B	1.00	1	1.00	1	1.00	1	1.00	1
C1 and C2	0.76	0.27; 2.15	0.82	0.27; 2.46	1.25	0.43; 3.59	1.01	0.32; 3.17
D and E	0.44	0.16; 1.19	0.40	0.13; 1.15	0.72	0.26; 1.98	0.45	0.15; 1.38
Number of people in the household								
≥ 4 people	1.00	1	1.00	1	1.00	1	1.00	1
< 4 people	1.54	0.86; 2.76	1.59	0.86; 2.95	2.04	1.17; 3.57	1.89	1.04; 3.46
Self‐perception of health^c^								
Very good/good	1.00	1	1.00	1	1.00	1	1.00	1
Fair/poor/very poor	2.27	1.26; 4.09	2.49	1.35; 4.58	4.23	2.40; 7.46	**4.12**	**2.28; 7.42**

*Note:* PrevOI Study (2020/2021). Crude and adjusted odds ratios (ORs) were presented with their respective 95% confidence intervals (95% CI), adopting a significance level of *p* < 0.05.

Abbreviation: OR, odds ratio.

^a^Multinomial logistic regression.

^b^95%CI = 95% confidence interval.

^c^Parent/guardian data. Notes: Model 1: adjusted for “sex,” “race/color,” “education,” “socioeconomic class,” and “number of people in the household.” Model 2: adjusted for “sex,” “race/color,” “education,” “socioeconomic class,” “number of people in the household,” and “self‐perception of health.”

When analyzing obesity, in the crude model, associations were observed with age ≥ 30 years, Black race/color skin, higher education, elementary school education, fewer than four people living in the household, and regular/poor/very poor self‐perceived health (Table 4). After adjustments, the variables that remained statistically significant were age ≥ 30 years, Black race/color skin, and regular/poor/very poor self‐perceived health (Table 4).

## 4. Discussion

This study observed concordance between the nutritional status of children and their parents/guardians, especially in the condition of obesity. A total of 56.5% of children with obesity also had parents/guardians with obesity. In addition, the likelihood of obesity among parents or caregivers was higher among individuals aged 30 years or older, of Black race/color skin, and with regular/poor/very poor self‐perceived health. These findings reflect the influence of environmental, sociodemographic, and health factors on obesity across the life course [7].

The formation of eating habits occurs mainly during the early years of life and tends to persist throughout life [23]. During this period, learning related to eating behavior, including the types of foods consumed, meal timing, and the contexts in which they occur, is influenced by parents’ practices, beliefs, and attitudes, transmitted from one generation to the next. This process results from the interaction between biological factors, characteristics of the family environment, and broader contextual conditions, which together shape children’s eating experiences [24].

The resemblance in nutritional status between children and their caregivers has been examined globally. A 2017 cross‐sectional study in China found weaker overall correlations, but stronger associations in children aged 6 –9 years, an age range predominantly cared for by mothers, compared to children aged 10–17 years. The correlation coefficients ranged from 0.30 to 0.35 in younger children and from 0.15 to 0.24 in older ones (*p* = 0.001), suggesting that family mealtimes and caregiver proximity influence these outcomes [7]. In our study, children aged 7–10 years were assessed, and the primary caregiver was also typically the mother, likely due to cultural norms around maternal caregiving.

In Brazil, there has been an alarming increase in overweight, particularly among children. Data from the Brazilian National Survey on Child Nutrition (ENANI‐2019) reported a higher prevalence of excess weight in mother–child dyads involving children under 5 years old (5.7%; 95% CI: 3.8–7.5), particularly in the southern region [8]. This can be attributed to shared genetic, behavioral, and contextual factors between mothers and children.

However, studies focusing on the association between the nutritional status of caregivers and children aged 6–10 years in the context of primary care are scarce in Brazil. One such study, conducted in Bragança Paulista in 2003, found a significant association between childhood obesity and having the mother as the main caregiver rather than the father (*p* = 0.014) [9]. These findings align with the present study and may reflect regional similarities in sociodemographic characteristics.

Obesity prevalence tends to increase with age [25]. Similarly, in the present study, adults aged ≥ 30 years had a higher likelihood of obesity. According to the Family Budget Survey (2007–2008), the highest prevalence of overweight occurred among individuals aged 45–54 and 55–64 years [25]. This may be due to increased time constraints, greater intake of ultra‐processed foods, and limited PA in this age group [26].

An ecological study conducted in Espírito Santo between 2009 and 2018 also showed increasing overweight trends among men and women, with distinctions by region and age [27]. These trends are shaped by various life events, such as pregnancy, hormonal changes, menopause, and accumulated lifestyle risks [28].

In Brazil, disparities in health and nutrition are deeply intertwined with race/color skin. Black individuals often face reduced access to healthcare and structural inequalities that directly affect nutritional outcomes [29]. In this study, Black race/color skin was associated with higher odds of obesity, a finding consistent with national data (POF 2008–2009) [29]. Notably, among women with higher socioeconomic status, Black individuals had a threefold higher likelihood of obesity compared to White ones. This may be explained by the psychosocial stress caused by racial inequality, which contributes to unhealthy eating behaviors and weight gain [10]. Worse self‐perceived health was another factor associated with obesity in our study. Individuals with obesity often experience body dissatisfaction, which can negatively impact health behaviors and self‐care [30]. Body image concerns, independent of socioeconomic status, have been shown to be strongly associated with obesity [29].

Obesity stigma also plays a critical role in shaping health perceptions. In a cross‐sectional study, 70.6% of participants reported at least one stigmatizing experience related to body weight, particularly among individuals with higher BMI. This stigma contributes to a poorer self‐assessment of health and can hinder realistic awareness of one’s health status, thereby impeding behavior change [31]. Thus, self‐perceived health, when classified as regular, poor, or very poor, may indirectly reflect children’s nutritional conditions [32].

Considering the multifactorial nature of obesity, several mechanisms have been proposed to explain its concurrent occurrence among parents and children. These mechanisms include both shared genetic inheritance and exposure to family environments with obesogenic characteristics. Evidence accumulated over recent decades, particularly from experimental studies, suggests the involvement of transgenerational epigenetic inheritance processes that increase susceptibility to obesity in subsequent generations [33, 34]. In this context, maternal obesity and exposure to overnutrition during pregnancy and the lactation period have been identified as key factors in the modulation of these epigenetic mechanisms [35, 36].

With regard to the obesogenic environment, defined as the set of environmental conditions that promote weight gain, such as the widespread availability of low‐nutritional‐quality foods and limited opportunities for regular PA, there remains a lack of consensus on the specific risk factors present within the family context, particularly with respect to their impact on body weight among children [37]. Nevertheless, evidence suggests that parental behaviors and practices constitute central components of this environment, given that parents serve as role models for their children [37] and that children are strongly influenced by the spaces and interactions to which they are most frequently exposed [38]. In addition, unmeasured sociobehavioral factors, including parental feeding practices and household routines, may play an important contextual role in shaping childhood weight outcomes. The absence of detailed information on these factors should be considered when interpreting the observed associations.

This study presents some limitations that should be considered when interpreting the results. The use of a convenience sample may have introduced selection bias and restricted the generalizability of the findings to other populations. However, the analysis focused primarily on investigating the association between parental and childhood obesity, prioritizing internal validity over population representativeness. Furthermore, the sample size achieved proved adequate for detecting associations of moderate to high magnitude. Nevertheless, caution is recommended when extrapolating the results, and future studies with designs and sampling strategies that enhance representativeness and external robustness are encouraged.

The cross‐sectional design of the study constitutes another significant limitation, as it does not allow for the establishment of causal relationships or the determination of the temporal sequence between exposures and the evaluated outcome. Thus, the findings should be interpreted as associations observed at a single point in time, and longitudinal studies are necessary to confirm temporal and causal links. In addition, it should be noted that some information was obtained through self‐reporting, particularly regarding breastfeeding history, which may be subject to recall bias. Consequently, results related to this variable should be interpreted with caution.

Also, this study has several strengths. Data were collected using standardized procedures and previously validated instruments, ensuring the quality and consistency of the information. The sample size was sufficient to provide adequate statistical power to detect associations of moderate to high magnitude. The study specifically focused on the associations between parental and childhood obesity, a topic of great public health relevance. Furthermore, the analysis considered multiple contextual factors, including socioeconomic, cultural, and environmental determinants. These findings provide practical insights for family‐based interventions and public policies aimed at preventing childhood obesity. Finally, the involvement of families (children, parents, and caregivers) regularly monitored by the FHS enhances the practical relevance of this study.

## 5. Conclusion

The nutritional status of the children with obesity aged 7–10 years was concordant with the obesity condition of their parents/guardians. In addition, in this study, the following factors were identified as being associated with parental/guardian obesity: being aged 30 years or older; self‐identifying as Black race/color skin; and having a self‐perceived health status classified as regular, poor, or very poor.

Socioeconomic, cultural, commercial, and environmental factors play a significant role in the prevalence of obesity among children and their guardians. Therefore, childhood obesity prevention efforts should be family‐based; planned within the healthcare system; and implemented in schools, recreational spaces, and community health services. These efforts should include lectures on healthy eating, cooking workshops, physical activities, and appropriate home routines led by qualified healthcare professionals.

Furthermore, public policies should be implemented to tackle obesity at all levels, including the promotion of adequate nutrition, self‐care regarding health, and the encouragement of PA within the context of primary healthcare.

## Author Contributions

Kéllyda Cinnara da Silva Moura: conceptualization, investigation, writing–original draft, methodology, visualization, writing–review and editing, formal analysis, and project administration. Oscar Geovanny Enriquez‐Martinez: writing–review and editing, supervision, and methodology. Haysla Xavier Martins Mendonça: writing–review and editing, formal analysis, and data curation. Leticia Batista de Azevedo: writing–review and editing, formal analysis, and data curation. Ana Maria Abreu de Oliveira: writing–review and editing, project administration, and investigation. Ana Luiza Leite Costa: writing–review and editing, project administration, and investigation. Pedro Antonio Souza de Almeida: project administration, investigation, and writing–review and editing. Maria del Carmen Bisi Molina: supervision, project administration, writing–review and editing, methodology, and funding acquisition.

## Funding

This study was supported by the National Council for Scientific and Technological Development (CNPq) (Process No. 443099/2020‐0); Maria del Carmen Bisi Molina, Espírito Santo Research and Innovation Support Foundation (FAPES) (Process No. 2021‐KT2Q4); and Kéllyda Cinnara da Silva Moura, scholarship holder, FAPES (Process No. 2021‐KT2Q4).

## Ethics Statement

The research followed the ethical standards of the Declaration of Helsinki for studies involving human beings and was approved by the Research Ethics Committee of the Health Sciences Center of the Federal University of Espírito Santo (protocol no 39633320.0.0000.5060/2020). Children signed the Free and Informed Assent Form, and the adults signed the Free and Informed Consent Form.

## Consent

Please see the Ethics Statement.

## Conflicts of Interest

The authors declare no conflicts of interest.

## Data Availability

Data are available on request due to privacy/ethical restrictions. The data that support the findings of this study are available on request from the corresponding author. The data are not publicly available due to privacy or ethical restrictions.
